# Reduced acute neuroinflammation and improved functional recovery after traumatic brain injury by α-linolenic acid supplementation in mice

**DOI:** 10.1186/s12974-016-0714-4

**Published:** 2016-09-23

**Authors:** Abhishek Desai, Taeyeop Park, Jaquel Barnes, Karl Kevala, Huazhen Chen, Hee-Yong Kim

**Affiliations:** Laboratory of Molecular Signaling, National Institute of Alcohol Abuse and Alcoholism, National Institutes of Health, 5625 Fishers Lane, Rm. 3N-07, Bethesda, MD 20892-9410 USA

**Keywords:** Polyunsaturated fatty acids (PUFA), Docosahexaenoic acid (DHA), Alpha-linolenic acid (ALA), Traumatic brain injury (TBI), Inflammation, Diet, Nutrition

## Abstract

**Background:**

Adequate consumption of polyunsaturated fatty acids (PUFA) is vital for normal development and functioning of the central nervous system. The long-chain n-3 PUFAs docosahexaenoic acid (DHA) and eicosapentaenoic acid are anti-inflammatory and neuroprotective in the models of central nervous system injury including traumatic brain injury (TBI). In the present study, we tested whether a higher brain DHA status in a mouse model on an adequate dietary α-linolenic acid (ALA) leads to reduced neuroinflammation and improved spontaneous recovery after TBI in comparison to a moderately lowered brain DHA status that can occur in humans.

**Methods:**

Mice reared on diets with differing ALA content were injured by a single cortical contusion impact. Change in the expression of inflammatory cytokines was measured, and cellular changes occurring after injury were analyzed by immunostaining for macrophage/microglia and astrocytes. Behavioral studies included rotarod and beam walk tests and contextual fear conditioning.

**Results:**

Marginal supply (0.04 %) of ALA as the sole dietary source of n-3 PUFA from early gestation produced reduction of brain DHA by 35 % in adult offspring mice in comparison to the mice on adequate ALA diet (3.1 %). The DHA-depleted group showed significantly increased TBI-induced expression of pro-inflammatory cytokines TNF-α, IL-1β, and IL-6 in the brain as well as slower functional recovery from motor deficits compared to the adequate ALA group. Despite the reduction of pro-inflammatory cytokine expression, adequate ALA diet did not significantly alter either microglia/macrophage density around the contusion site or the relative M1/M2 phenotype. However, the glial fibrillary acidic protein immunoreactivity was reduced in the injured cerebral cortex of the mice on adequate ALA diet, indicating that astrocyte activation may have contributed to the observed differences in cellular and behavioral responses to TBI.

**Conclusions:**

Increasing the brain DHA level even from a moderately DHA-depleted state can reduce neuroinflammation and improve functional recovery after TBI, suggesting possible improvement of functional outcome by increasing dietary n-3 PUFA in human TBI.

## Background

Modern dietary practice has resulted in an increased consumption of n-6 polyunsaturated fatty acids (PUFA) with a corresponding deficit in n-3 PUFA. This phenomenon has skewed the n-6 to n-3 PUFA ratio, which is about 15:1 in modern diets [1]. Neuronal membranes preferentially incorporate docosahexaenoic acid (DHA; 22:6n-3), and its deficiency leads to altered gene expression in the mouse brain [2]. As the brain tissue content of DHA depends on the amount of n-3 PUFA in the diet, decreased n-3 PUFA consumption would translate to DHA deficiency in the brain. DHA deficiency results in increased n-6 docosapentaenoic acid (DPAn-6; 22:5n-6) in the brain as a substitute for DHA. Mice reared on adequate n-3 PUFA diet have very low levels of DPAn-6 in the brain (with the approximate DPAn-6 to DHA ratio less than 0.01), which increases in mice on low n-3 PUFA diet with a corresponding decrease in DHA [3]. Therefore, the relative concentration of DPAn-6 in the neural membrane may be used as an index of DHA deficiency. Interestingly, significant amount of DPAn-6 has been detected in the human brain with the DPAn-6 to DHA ratio in the phospholipids of about 0.44 [4]. Assuming that the DHA/DPAn-6 proportion in mice extrapolates to humans, this indicates that modern human brains may not have the optimum DHA levels.

In addition to their role in development, n-3 PUFA, especially DHA and eicosapentaenoic acid (EPA; 20:5n-3), have been shown to regulate immunity [5]. Dysregulated inflammation is an important pathological factor in models of central nervous system injury. Inflammatory responses in the brain have been attributed to local cellular responses wherein glial cells, in particular, secrete pro-inflammatory cytokines and chemokines. Under the influence of chemokines, there is an influx of neutrophils and monocytes/macrophages into the brain tissue parenchyma facilitated by a state of non-selective permeability brought about by the disruption of the blood-brain barrier [6, 7]. The invading neutrophils, macrophages, and resident glial cells produce both pro- and anti-inflammatory cytokines such as interleukin (IL)-1β, tumor necrosis factor (TNF)-α, IL-6, and IL-10 [8].

Pre-treatment with n-3 PUFA confers resilience and protects against brain injury [9–11]. Administration of these fatty acids has also been reported to be neuroprotective after stroke [12] and reduce axonal damage after spinal cord injury [13, 14] and head injury [15]. We recently reported impaired recovery from traumatic brain injury (TBI) in mice that were severely depleted in brain DHA (~70 %) [16]. DHA deficiencies of this magnitude are not expected to be present in humans. However, brain DHA levels can be lower if the n-3 PUFA intake is insufficient, as in the case of infants fed with formula milk lacking n-3 PUFA showing lower levels of brain DHA than those on breast milk [17, 18]. In this study, we sought to generate higher DPAn-6 to DHA ratio in mouse brains similar to that in humans by providing low level of α-linolenic acid (ALA; 18:3n-3) in the diet for 4 months from late gestation. Their cellular and behavioral responses to TBI were compared to those of mice on adequate ALA diet which resulted in higher levels of brain DHA and only trace amount of DPAn-6.

## Methods

### A mouse model of low and adequate n-3 PUFA

Pregnant (E14) C57BL6/N mice purchased from Charles River were placed on a diet containing a low ALA content (0.04 %). The adequate n-3 PUFA group received a diet containing flaxseed oil as a source of ALA (3.1 % ALA) (Table 1). The fatty acid content of the diet is shown in Table 2. Male offspring were reared on the same low or adequate ALA diet till 4 months of age. The normal 12-h light period was maintained in the animal housing facility, and mice had free access to food and water.

**Table 1:** Fatty acid sources in diets

Lipid source	Low ALA diet (g/kg)	Adequate ALA diet (g/kg)
Tocopherol-stripped safflower oil	19	17.7
Flaxseed oil	0	4.81
Hydrogenated coconut oil	81	77.49

**Table 2:** Fatty acid content of diets

Fatty acid	Low ALA diet (weight %)	Adequate ALA diet (weight %)
Lauric acid (12:0)	36.06 ± 0.80	35.29 ± 1.61
Myristic acid (14:0)	17.90 ± 0.17	16.64 ± 0.15
Palmitic acid (16:0)	11.35 ± 0.09	10.90 ± 0.19
Stearic acid (18:0)	12.06 ± 0.07	11.71 ± 0.20
Oleic acid (18:1n9)	3.85 ± 0.06	4.47 ± 0.16
Vaccenic acid (18:1n7)	0.26 ± 0.00	0.27 ± 0.01
Linoleic acid (18:2n6)	18.19 ± 0.47	17.19 ± 0.68
γ-Linolenic acid (18:3n6)	N.D.	0.07 ± 0.06
Arachidonic acid (20:4n6)	N.D.	N.D.
α-Linolenic acid (18:3n3)	0.04 ± 0.04	3.08 ± 0.15
Docosahexaenoic acid (22:6n3)	N.D.	N.D.

Data are expressed as mean ± SD (*n* = 3)

*N.D.* not detected

### TBI model

A single controlled cortical impact was delivered to the exposed brain as previously reported [16]. Mice were anesthetized with 5 % isoflurane and then maintained under anesthesia at 2.5–3 % isoflurane during surgery. The mouse head was fixed in a stereotaxic apparatus. A ~4 mm craniotomy was made with a drill over the left cerebral hemisphere between the bregma and lambda and the stereotaxic apparatus angled to make the plane of the dura perpendicular to the impact. The flat-tipped 3-mm-diameter pin of the precision TBI head impactor delivered a pneumatically controlled impact at 3.5 m/s velocity with 1.5 mm penetration. The injury site was covered with Surgicel and the craniotomy sealed with a plastic cap glued using cyanoacrylate. The incision was glued and a topical analgesic cream (EMLA) was applied for analgesia. Lidocaine jelly was instilled in the ears, and the mouse was placed in a cage over a hot water blanket at 37 °C.

### Fatty acid analysis

The contralateral cerebral hemisphere of the brains from 5-month-old mice was homogenized in 1:1 volumes of methanol/butylated hydroxytoluene and Tris buffer (pH 7.4) for lipid extraction by the Bligh-Dyer method [19]. Chloroform and water were added to the homogenate to attain a chloroform to methanol to water ratio of 2:2:1.8. The homogenate was vortexed after displacing the air by nitrogen to prevent oxidation and centrifuged at 3000 rpm at 4 °C. The organic layers were collected, and the aqueous layer was treated for repeated extraction as above. The lipids were transmethylated at 100 °C for 2 h under nitrogen using borontrifluoride and methanol, and fatty acids were analyzed by gas chromatography [20].

### qRT-PCR

At 4 h, 24 h, or 4 days after TBI, mice were deeply anesthetized with isoflurane and perfused quickly with chilled phosphate-buffered saline under continued anesthesia. The brain was removed, and the peri-contusional cortex was dissected and immediately immersed in RNAlater solution to preserve RNA integrity. The RNA was isolated using TRIzol and reverse transcribed using Applied Biosystems cDNA Reverse Transcription kit (Fisher Scientific, Waltham, MA, USA) and amplified using QuantiTect SYBR PCR kit (Qiagen, Valencia, CA, USA). Glyceraldehyde-3-phosphate dehydrogenase (GAPDH) was used as an internal control to normalize the gene expression levels. The gene expression changes are displayed relative to the respective control (sham) level.

Primer sequences were as follows:

TNF-α: forward 5′-CCCTCCAGAAAAGACACCATG-3′, reverse 5′-GCCACAAGCAGGAATGAGAAG-3′

IL-1β: forward 5′-CCACCTTTTGACAGTGATGA-3′, reverse 5′-GAGATTTGAAGCTGGATGCT-3′

IL-6: forward 5′-GTCGGAGGCTTAATTACACA-3′, reverse 5′-TTTTCTGCAAGTGCATCATC-3′

IL-10: forward 5′-AGCCTTATCGGAAATGATCC-3′, reverse 5′-GGGAATTCAAATGCTCCTTG-3′

Chemokine (C–C motif) ligand 2 (CCL2): forward 5′-GGATCGGAACCAAATGAGAT-3′, reverse 5′-ATTTACGGGTCAACTTCACA-3′

Cluster of differentiation (CD)-16: forward 5′-TTTGGACACCCAGATGTTTCAG-3′, reverse 5′-GTCTTCCTTGAGCACCTGGATC-3′

CD-32: forward 5′-AATCCTGCCGTTCCTACTGATC-3′, reverse 5′-GTGTCACCGTGTCTTCCTTGAG-3′

CD-206: forward 5′-CAAGGAAGGTTGGCATTTGT-3′, reverse 5′-CCTTTCAGTCCTTTGCAAGC-3′

Arg1: forward 5′-CTATGTGTCATTTGGGTGGA-3′, reverse 5′-TCTGGGAACTTTCCTTTCAG-3′

Ym1/2: forward 5′-CAGGGTAATGAGTGGGTTGG-3′, reverse 5′-CACGGCACCTCCTAAATTGT-3′

GAPDH: forward 5′-CCACTCACGGCAAATTCAAC-3′, reverse 5′-CTCCACGACATACTCAGCAC-3′

### Protein expression

Mice were perfused with chilled phosphate-buffered saline (PBS) at 4 h, 24 h, and 4 days after TBI. The peri-contusional cortex was carefully dissected and homogenized in Tris-based lysis buffer containing protease inhibitors, sonicated and centrifuged at 4 °C at 12,000 rpm for 15 min. The supernatant was collected and stored at −80 °C until analysis. A commercial CCL2 ELISA kit (Raybiotech, Norcross, GA, USA), which is a colorimetry-based sandwich ELISA kit, was used to quantify the CCL2 protein expression according to the manufacturer’s instructions. For the western blotting, samples were run in pre-formed 4–12 % gradient gels, transferred to polivinylidene difluride membrane, and incubated with mouse anti-IL-1β antibody (Cell Signaling Technology, Danvers, MA, USA). Bands were visualized and quantified to assess the relative changes in expression of IL-1β using a Kodak Gel Logic 440 imaging system with ImageQuant 5.1 software (Molecular Dynamics, Sunnyvale, CA).

### Immunofluorescence

At 3 days after TBI, the injured mice were perfused with chilled PBS and then with 4 % paraformaldehyde. The brains were immersed in paraformaldehyde for 24 h and subsequently dehydrated with ethanol and permeated/embedded with paraffin. Six-micrometer-thick sections were cut with a cryotome and mounted on charged slides. Four sections from the injury epicenter for each brain were later deparaffinized, subjected to antigen retrieval by boiling in citrate buffer for 20 min, and blocked with 5 % goat serum for 1 h at room temperature. They were then incubated overnight at 4 °C in anti-ionized calcium-binding adaptor molecule 1 (Iba-1) (catalog number 019-19741; Wako Chemicals, Richmond, Virginia, USA) or anti-glial fibrillary acidic protein (GFAP) antibody (catalog number G9269; Sigma-Aldrich, Saint Louis, MO, USA) solution followed by secondary antibody tagged with Alexa Fluor 488 (Thermo Fisher, Waltham, MA, USA). The fluorescence was visualized with a ×20 objective and quantified using the MetaMorph software (Molecular Devices Inc., Sunnyvale, CA, USA). Cells were counted in the peri-contusional cortex in three non-overlapping fields per section and the corresponding fields for the contralateral cortex.

### Motor function tests

Motor deficits were assessed using the rotarod and beam walk tests. Mice were trained to balance on an accelerating rotarod (0 to 400 rpm) for 5 min. The training schedule began 4 days prior to the day of surgery and consisted of three trials each day for 3 days with the baseline performance recorded on the fourth day. The mice were tested daily for 6 days after surgery, and the average latency to fall over three trials on each day was recorded. The beam walk test consisted of walking across a 50-cm-long 0.7-cm-wide beam. The mice were trained for 2 days with three trials per day followed by recording the baseline performance on the day preceding surgery. The total hind limb steps and foot slips were counted on the day before surgery. The test was conducted daily after surgery for 7 days.

### Fear conditioning

The fear conditioning experiment was performed over 3 days starting 3 weeks after injury with the first day serving as the day of habituation, the second as the day of fear conditioning, and the third as the day of testing the memory. Mice were individually placed inside the fear conditioning chamber (Freeze Monitor, San Diego Instruments, San Diego, CA, USA) on the first day and allowed to explore for 5 min. On the following day, mice were given two shocks at 120 and 150 s after being introduced into the fear conditioning chamber and were taken out of the chamber 60 s after the last shock. Each shock was 0.5 mA and lasted for 0.5 s. The mice were tested for fear memory 24 h after the fear conditioning session by placing them again in the fear conditioning chamber and assessing freezing for 5 min. Freezing in mice was defined as the absence of any movement except for breathing. Freezing was also monitored on the first day to ascertain baseline freezing.

### Statistical analyses

The data for behavioral tests were analyzed using repeated measures two-way analysis of variance (ANOVA) with Prism 6 for Windows (GraphPad software Inc., La Jolla, CA, USA). Multiple comparisons between experimental groups were made using Fisher’s least significant difference (LSD) test. Two-way ANOVA was also performed for cytokine expression and microglial phenotype analyses; Sidak’s multiple comparisons test was used for comparing individual groups. Groups were compared for the fatty acid analysis and astroglia immunofluorescence by unpaired two-tailed Student’s *t* test.

## Results

### Mice on adequate ALA diet have higher DHA levels in the brain

**Fig. 1:**
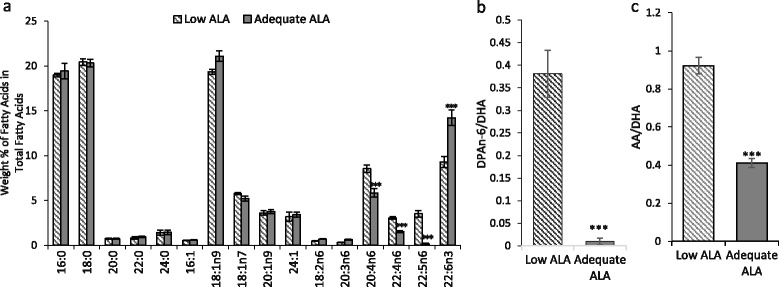
Dietary α-linolenic acid increases brain docosahexaenoic acid content. **a** Lipid analysis of the brain samples of mice revealed that mice on adequate α-linolenic acid (ALA) diet had more docosahexaenoic acid (DHA; 22:6n-3), less arachidonic acid (AA; 20:4n-6), n-6 docosatetraenoic acid (22:4n-6), and n-6 docosapentaenoic acid (DPAn-6; 22:5n-6) in the brain compared to mice on low ALA diet. **b**, **c** Mice on adequate ALA diet had lower ratio of DPAn-6 to DHA (**b**) as well as AA to DHA (**c**) compared to low ALA diet mice. *n* = 4 each. ***p < 0.001 vs low ALA diet group. Data are expressed as mean ± SD

Higher levels of DHA, an endogenous product of ALA, were found in the brain of mice on the diet having 3.1 % ALA (Fig. 1) compared to those on the low ALA diet. The average value of DHA was 50 % higher in the mice on adequate ALA diet (14.23 ± 0.87 %) than in the mice on low ALA diet (9.29 ± 0.64 %) (a). The increase in DHA was mirrored by a corresponding decrease in n-6 PUFAs including arachidonic acid (AA; 20:4n-6) (8.54 ± 0.41 % vs 5.85 ± 0.45 %), n-6 docosatetraenoic acid (22:4n-6) (3.03 ± 0.14 % vs 1.53 ± 0.12 %), and DPAn-6. The DPAn-6 level decreased from 3.51 ± 0.3 % in the brain of low ALA diet mice to a nearly undetectable level in the mice on adequate ALA diet. As a consequence, the adequate ALA diet reduced the DPAn-6 to DHA ratio from 0.38 ± to 0.05 to 0.01 ± 0.007 % (b) and the AA to DHA ratio from 0.92 ± 0.04 to 0.41 ± 0.02 % (c).

### Mice with higher brain DHA show less neuroinflammation after TBI

**Fig. 2:**
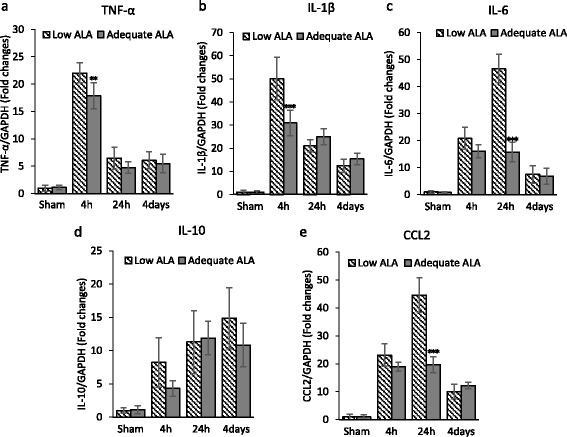
**a**–**e** Suppression of TBI-induced acute inflammatory cytokine expression in mice with higher brain docosahexaenoic acid. TBI induced the gene expression of pro-inflammatory cytokines in the cerebral cortex. Adequate α-linolenic acid (ALA) diet resulted in lower expression of pro-inflammatory cytokines TNF-α, IL-1β, IL-6, and CCL2 after TBI as compared to the low ALA group. *n* = 3–4. ***p < 0.001, **p < 0.01 compared to the low ALA diet group for the respective time point. Data are expressed as mean ± SD

**Fig. 3:**
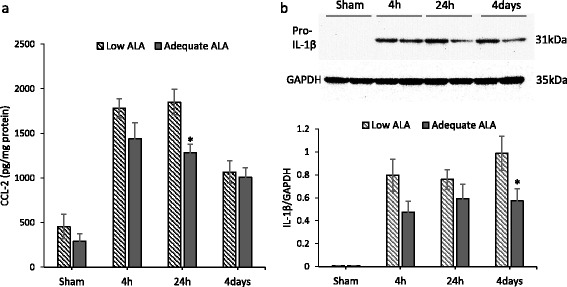
**a**, **b** Suppression of traumatic brain injury-induced CCL2 and IL-1β protein expression in mice with higher brain docosahexaenoic acid. Traumatic brain injury caused induction of CCL2 and IL-1β protein expression in the cerebral cortex at the site of injury. The expression of both proteins was lower in mice on adequate α-linolenic acid (ALA) diet compared to the low ALA group. *N* = 3–4. *p < 0.05 compared to the low ALA diet group for the respective time points. Data are expressed as mean ± SEM

The expression of pro-inflammatory cytokines TNF-α, IL-1β, IL-6, CCL2, and the anti-inflammatory cytokine IL-10 in the DHA-low and DHA-high brains was measured at 4 h, 24 h, and 4 days after TBI. TBI induced an increase in all pro-inflammatory cytokines examined. Two-way ANOVA revealed a significant elevation in the expression of TNF-α (*p* < 0.0001), IL-1β (*p* < 0.0001), IL-6 (*p* < 0.0001), and CCL2 (*p* = 0.0008) after TBI (Fig. 2). The TBI-induced production of these cytokines was affected by the brain DHA status. Sidak’s multiple comparisons test revealed significantly lower expression of TNF-α (22.0 ± 1.8 vs 17.8 ± 2.4; *p* = 0.007) as well as IL-1β transcript levels (50.0 ± 9.2 standard deviation (SD) vs 30.9 ± 5.5; *p* < 0.0001) in the adequate ALA diet group at 4 h after TBI. Similar differences were present for IL-6 (46.7 ± 5.3 vs 15.7 ± 3.6; *p* < 0.0001) and CCL2 (44.6 ± 6.2 vs 19.7 ± 2.9; *p* < 0.0001) at 24 h after TBI. The expression of the anti-inflammatory cytokine IL-10, despite its elevation after TBI, was not significantly affected by diet for any of the time points considered in this study. To evaluate changes in protein expression, the CCL2 and IL-1β expression was assessed by ELISA and western blotting, respectively. CCL2 and IL-1β protein expression increased after TBI and remained elevated at all time points examined (Fig. 3). Two-way ANOVA showed significant effects for injury (*p* < 0.001) and diet (*p* < 0.01) for both the cytokines. Sidak’s multiple comparisons test revealed a significant decrease in CCL2 expression at 24 h post-injury for mice on adequate ALA diet compared to mice on low ALA diet (*p* < 0.05) (Fig. 3a). The expression of IL-1β protein was significantly lower at 4 days after injury for the adequate ALA diet mice compared to those on low ALA diet (*p* < 0.05) (Fig. 3b).

### Acute post-injury astrogliosis is modulated by n-3 PUFA status without affecting microglial density

**Fig. 4:**
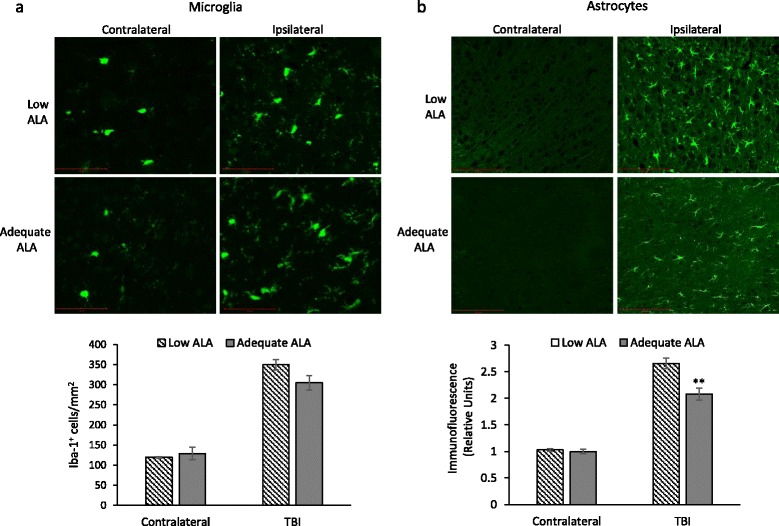
Glial reactivity in mice on low or adequate α-linolenic acid diet after traumatic brain injury. **a** Traumatic brain injury (TBI) resulted in distinct increase in Iba-1-positive cells in the peri-contusional cortex at 3 days post-injury. Diets producing different brain docosahexaenoic acid (DHA) contents did not affect the density of Iba-1-positive cells at the injury site. **b** Cortical GFAP immunofluorescence increased 3 days after TBI was more in mice on low α-linolenic acid (ALA) diet that have less brain DHA than the adequate ALA group. *n* = 3–4. **p < 0.01 vs control TBI. Data are expressed as mean ± SEM. *Scale bar* 100 μm

Iba-1 is expressed by microglia and macrophages. TBI resulted in a threefold increase in the number of Iba-1-positive cells around the contusion site compared to the contralateral cortex (Fig. 4a). However, there was no significant difference in the number of Iba-1-positive cells between the diet groups. The mean Iba-1-positive cell count obtained from the contralateral cortex was 119.4 ± 2.0 and 129.1 ± 15.9 for the low ALA and adequate ALA diet group, respectively. In the injured mouse brains, mean counts of Iba-1-positive cells accumulated around the injury site were 349.3 ± 13.0 and 304.8 ± 17.7 for the low ALA and adequate ALA diet groups, respectively. GFAP immunoreactivity in the contralateral cortex was negligible (Fig. 4b). However, TBI induced a robust increase in GFAP immunofluorescence, indicating astrocyte activation. Mice with DHA deficiency showed higher GFAP immunofluorescence in the injured cortex (*p* < 0.01) compared to the mice that had greater DHA (2.6 ± 0.1 vs 2.1 ± 0.1, respectively).

### Altering the brain n-3 PUFA has limited effect on microglial M1/M2 polarization

**Fig. 5:**
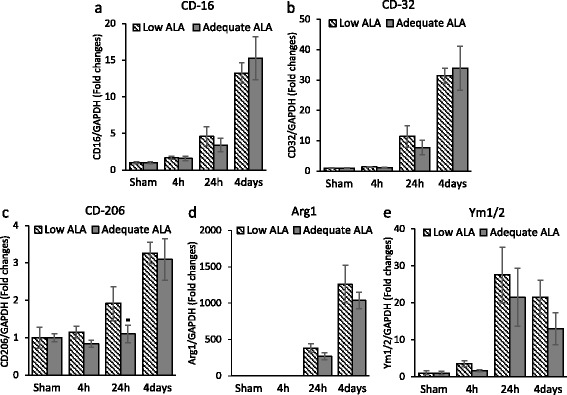
Diets differing in α-linolenic acid content have limited effects on microglial polarization. The expression of M1 microglia/macrophage markers CD-16 and CD-32 (**a**, **b**) and M2 microglia/macrophage markers CD-206, Arg-1, and Ym1/2 (**c**-**e**) was increased in peri-contusional cortex after traumatic brain injury. Dietary α-linolenic acid (ALA) content showed no effect on the expression of these markers in general, with the exception of CD-206 at 24 h after TBI. *n* = 3–4. *p < 0.05 vs low ALA at the same time points. Data are expressed as mean ± SD

Since the microglial response to injury depends on their phenotype, we assessed the expression of CD-16 and CD-32 for M1 microglia and CD-206, arginase 1 (Arg-1), and Ym1/2 for M2 microglia. TBI induced distinct increases in the expression of all these markers, particularly from 24 h (Fig. 5). However, with the sole exception of CD-206, there was no statistically significant change in the relative expression of these M1/M2 markers due to n-3 PUFA differences. The expression of CD-206 was higher in the low ALA diet group (*p* < 0.05) (1.9 ± 0.1) at 24 h after TBI compared to the adequate ALA group (1.1 ± 0.2).

### Mice with more brain DHA exhibit better motor recovery after TBI

**Fig. 6:**
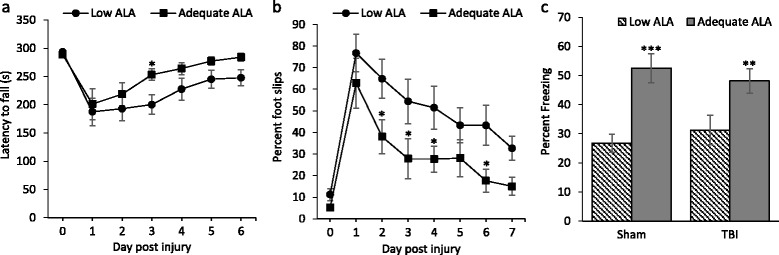
Effect of low or adequate α-linolenic acid diet on motor and cognitive changes after TBI. **a**, **b** Impaired motor performance in the rotarod (**a**) and beam walk tests (**b**) due to TBI was recovered faster in mice on adequate α-linolenic acid (ALA) diet compared to those on the low ALA diet that had less brain DHA. **c** Mice on adequate ALA diet showed more freezing after contextual fear conditioning compared to the mice on low ALA diet for both sham and TBI mice. ***p < 0.001, **p < 0.01, *p < 0.05 compared to the respective low ALA diet group. Data are expressed as mean ± SEM. *n* = 8–14

TBI resulted in motor deficits observed in the rotarod as well as beam walk tests (Fig. 6). Repeated two-way ANOVA revealed a significant effect of diet on the latency to fall off the rotarod (*p* < 0.01). The motor performance of all mice recovered gradually with time. The mean time on the rotarod for the mice on low ALA diet, and thus with lower brain DHA, did not reach the pre-injury levels even on the sixth day after injury (248 s ± 14.5 standard error of mean (SEM) compared to the pre-injury time of 293 s ± 2 SEM) (Fig. 6a). However, the performance of the mice on adequate ALA diet that have more brain DHA was comparable to the pre-injury level (288.6 ± 5.1 SEM) by the fifth day (277.2 ± 7.6 SEM). In the beam walk test, the mean values for foot slips for the adequate ALA diet mice remained lower than those of the low ALA group throughout the test period as shown by the significant difference on day 2, 3, 4, and 6 after injury (*p* < 0.05) (Fig. 6b).

Contextual fear conditioning test was used to assess differences in fear memory after TBI. The mice on adequate ALA diet had significantly better memory 24 h after the fear conditioning for both sham (*p* < 0.001) and TBI animals (*p* < 0.01) compared to the low ALA group as indicated by more freezing (Fig. 6c). However, TBI by itself did not affect freezing.

## Discussion

The present study investigated the effects of altering the brain DHA status on acute TBI outcome. The DHA level in mouse brains was modulated by differing levels of dietary ALA from late gestation to the adult stage. A moderately lower DHA level in the brain achieved by a diet lacking DHA and having low ALA was to model human brains with conspicuous presence of DPAn-6 [4]. With this diet, DPAn-6 was elevated in mouse brains with the DPAn-6 to DHA ratio of 0.38 (Fig. 1) which is similar to that observed in human brains (0.44) [4]. The brain DPA to DHA ratio below 0.01 that is normally observed in an n-3 PUFA-sufficient condition [3] was also achieved by providing 3.1 % ALA in diet.

Acute increase in inflammatory mediators is a part of fundamental tissue response to injury. Decrease in n-3 PUFA levels with a corresponding increase in the n-6 to n-3 PUFA ratio can lead to increase in inflammation [21]. Phospholipase A2 is activated after TBI [22], which elevates free fatty acid levels [23], leading to greater local availability of DHA and AA. While eicosanoids generated from AA can be neurotoxic under pathological conditions, docosanoids derived from DHA are often anti-inflammatory. Having higher DHA levels in the brain can lead to greater local free DHA levels after injury as well as more anti-inflammatory DHA metabolites. These attributes likely decreased acute inflammatory cytokine expression after TBI in mice with higher brain DHA (Fig. 2). The transcription factor nuclear factor kappa light chain enhancer of activated B cells (NF-kB) that upregulates the expression of pro-inflammatory genes has been shown to be activated after TBI [24]. In contrast, DHA/EPA supplementation can reduce NF-kB activity and inflammation, as shown for neonatal hypoxia-ischemia [9] and LPS-stimulated macrophages [25]. In addition, increase in oxidative stress in the injured brain leads to generation of oxidation products of n-3 PUFA, and these products can inhibit NF-kB by activating peroxisome proliferator-activated receptor (PPAR)α [26]. Thus, it is likely that raising the brain DHA level reduced pro-inflammatory cytokines by affecting the activity of these transcription factors.

ALA-rich diet was reported to lower the production of IL-6, IL-1β, and TNF-α by peripheral blood mononuclear cells of hypercholesterolemic subjects compared to controls fed with either high linoleic acid diet or a typical American diet [27]. These cytokines were also associated with injury-induced activation of not only macrophage/microglia but also astrocytes in the brain [28]. Pu et al. [11] have reported increase in pro-inflammatory cytokine expression along with an increase in microglia/macrophage count in n-3 PUFA-deficient mice after TBI. Our results are in agreement with the local increase in pro-inflammatory cytokines (Figs. 2 and 3). However, we did not find significant differences in the macrophage/microglial density around the injury site for the two diet groups (Fig. 4a). This may be a result of the differences in dietary n-3 PUFA used in the two studies. While we provided n-3 PUFA in the form of ALA in diet, Pu et al. [11] used fish oil (1.5 % DHA and EPA). This may change the blood levels of DHA/EPA that can impact the microglia activation after TBI. In addition, we had the mice on diet since late gestation, while in the study by Pu and colleagues, the mice were placed on diet at 3 weeks age. This is probably the reason for greater differences in brain DHA (about 35 % change) in our experiment as compared to the small but statistically significant changes of 10–15 % in the reported study. Furthermore, the DPAn-6 levels change dramatically with our dietary regiment but do not change in the case of Pu et al.’s study [11]. This is expected as the brain undergoes rapid accretion of DHA during early development when our dietary regimen was initiated while its levels recover at a slower rate after adolescence [3]. These factors may also account for the temporal differences in cytokine expression in these studies. Pu et al. assessed cytokine expression at 24 h post-injury and found significant differences between the two diet groups for TNF-α and IL-1β, while we found maximum expression and significant differences for these cytokines at 4 h in our time course analysis (Fig. 2). Nevertheless, we did observe changes in CCL2 and IL-1β protein expression at 24 h and 4 days, respectively (Fig. 3). Although we did not find a significant difference in the macrophage/microglial density near the injury site, there may also exist qualitative changes in these cells priming them toward a pro- or anti-inflammatory phenotype. The activated microglia/macrophages are categorized into a classical activated state or M1 and an alternate activated state or M2. M1 microglia/macrophages typically have a pro-inflammatory phenotype producing pro-inflammatory cytokines, having increased interaction with other immune cells, and contributing to oxidative/nitrosative stress [29]. On the other hand, M2 microglia/macrophages modulate phagocytosis and promote wound healing [29, 30]. We did not find any significant change in the M1/M2 phenotype marker expression for activated macrophage/microglia in the injured diet groups (Fig. 5). These findings suggest that the DHA-derived changes in inflammation may not be attributed to microglial activation at least in our experimental settings using ALA as the n-3 PUFA source.

Our data indicated increased induction of astrogliosis after TBI (Fig. 4b). Astrocyte activation is a complex process that can aid in limiting inflammation [31]. However, it can also lead to localized pro-inflammatory environment. For example, activation of astrocytes by TLR4 stimulation can lead to pro-inflammatory cytokine expression [32]. In contrast to microglial activation, astrogliosis was significantly increased in the DHA-depleted mouse brain. CCL2, which is mostly produced by astrocytes in the brain [33, 34], was significantly increased at 4 h after injury, indicating early activation of astrocytes. The brains from the mice on ALA adequate diet showed significantly reduced CCL2 at 24 h after injury compared to the low ALA diet group. It is possible that suppressed astrocyte activation with an increase in brain DHA in the adequate ALA diet mice (Fig. 4b) may have contributed to the reduction in CCL2 at 24 h of injury. DHA inhibits endoplasmic reticulum stress in astrocytes in the in vitro ischemia model [35] and reduces endoplasmic reticulum stress after TBI [36]. Thus, reduction in endoplasmic reticulum stress in the mice on adequate ALA diet may have reduced astrocyte activation after TBI.

Severe DHA depletion in the brain brought about by multi-generational n-3 PUFA dietary restriction causes significant impairment in functional recovery after TBI [16]. Similarly, a relatively moderate lowering in brain DHA levels also impaired motor recovery from TBI (Fig. 6a, b). It is possible that neuroinflammation aggravated by DHA deficiency contributed to the worse recovery outcome, although causal relationship still needs to be established. The increase in brain DHA can be responsible for the increase in contextual fear learning/memory (Fig. 6c) as DHA/n-3 PUFA is known to improve cognition, although this parameter was not responsive to TBI.

## Conclusions

Using a mouse model with brain DHA depletion, we demonstrate that increasing brain DHA can limit post-injury inflammation, reduce astrocyte activation, and improve functional recovery. This study suggests possible improvement of functional outcome by increasing brain DHA through diet in human TBI.

## Abbreviations

ALA: α-linolenic acid
ANOVA: Analysis of variance
Arg-1: Arginase 1
CD: Cluster of differentiation
CCL2: Chemokine (C–C motif) ligand 2
DHA: Docosahexaenoic acid
DPAn-6: Docosapentaenoic acid
EPA: Eicosapentaenoic acid
GFAP: Glial fibrillary acidic protein
Iba-1: Ionized calcium-binding adaptor molecule 1
IL: Interleukin
LSD: Least significant difference
NF-kB: Nuclear factor kappa light chain enhancer of activated B cells
PPAR: Peroxisome proliferator-activated receptor
PUFA: Polyunsaturated fatty acid
SD: Standard deviation
SEM: Standard error of mean
TBI: Traumatic brain injury
TNF: Tumor necrosis factor
